# Robotic resection of ectopic mediastinal parathyroid adenoma in multiple endocrine neoplasia 1

**DOI:** 10.1186/s40792-023-01694-9

**Published:** 2023-06-21

**Authors:** Yuko Ohara, Yuka Kadomatsu, Toyone Kikumori, Toyofumi F. Chen-Yoshikawa

**Affiliations:** 1 Department of Thoracic Surgery, Nagoya University Graduate School of Medicine, 65 Tsurumai-Cho, Showa-Ku, Nagoya, 466-8560 Japan; 2 Department of Breast and Endocrine Surgery, Nagoya University Graduate School of Medicine, Nagoya, Japan

**Keywords:** Ectopic parathyroid adenoma, Hyperparathyroidism, Multiple endocrine neoplasia 1, Robot-assisted thoracoscopic surgery

## Abstract

**Background:**

Hyperparathyroidism in patients with multiple endocrine neoplasia 1 is attributed to the excessive secretion of parathyroid hormone (PTH) from multiple parathyroid glands. This can be successfully treated through complete resection of the parathyroid glands; however, subsequent surgery is often required because of the presence of supernumerary or ectopic parathyroid glands. Therefore, identifying the locations of all functional glands is crucial for precise resection. Here, we report a case of ectopic mediastinal parathyroid adenoma that was successfully resected using robot-assisted thoracoscopic surgery.

**Case presentation:**

A 53-year-old woman underwent a total parathyroidectomy with autotransplantation for multiple endocrine neoplasia 1-associated primary hyperparathyroidism. The patient previously underwent laparoscopic distal pancreatectomy for a pancreatic neuroendocrine tumor. She also presented with a mediastinal tumor and nonfunctional pituitary adenoma that could be followed up. Blood tests before total parathyroidectomy showed high levels of intact PTH (183 pg/mL) and calcium (Ca; 10.3 mg/dL); however, postoperative blood tests still revealed high levels of intact PTH (103 pg/mL) and Ca (11.4 mg/dL). Computed tomography and magnetic resonance imaging revealed a 45-mm-sized mass in the right upper mediastinum as a well-defined solid and cystic lesion, whereas ^99m^Tc-methoxyisobutylisonitrile scintigraphy indicated a strong accumulation of tracers, suggesting an ectopic lesion in the mediastinum. Persistent hyperparathyroidism after total parathyroidectomy via neck incision was attributed to an ectopic parathyroid tumor in the mediastinum. Thus, we decided to resect the tumor using robot-assisted thoracoscopic surgery to perform the procedure gently and carefully. During surgery, a mediastinal tumor was identified as it was detected radiographically. Because it did not invade the surrounding tissues, it could be completely resected without injuring the capsule. The patient was discharged without complications. Postoperatively, Ca and intact PTH levels decreased back to normal. The final pathological diagnosis confirmed that the mass was an ectopic mediastinal parathyroid adenoma.

**Conclusions:**

Minimally invasive surgical resection of a remnant ectopic lesion was successfully performed in a patient with multiple endocrine neoplasia 1 using robot-assisted thoracoscopic surgery.

## Background

Hyperparathyroidism is characterized by an excessive secretion of parathyroid hormone (PTH) from the parathyroid glands, necessitating complete resection of the causative glands for successful treatment. This condition is also known to occur in patients with multiple endocrine neoplasia type 1 (MEN1) and often requires multiple subsequent surgeries because of the presence of supernumerary or ectopic parathyroid glands. Therefore, it is vital to identify the locations of all functional glands for successful resection.

Minimally invasive thoracoscopic surgery is performed worldwide to treat various diseases [1] and includes techniques, such as video- and robot-assisted thoracoscopic surgery (RATS). Herein, we report the case of a patient with a remnant ectopic mediastinal parathyroid adenoma who successfully underwent complete resection using RATS.

## Case presentation

**Fig. 1:**
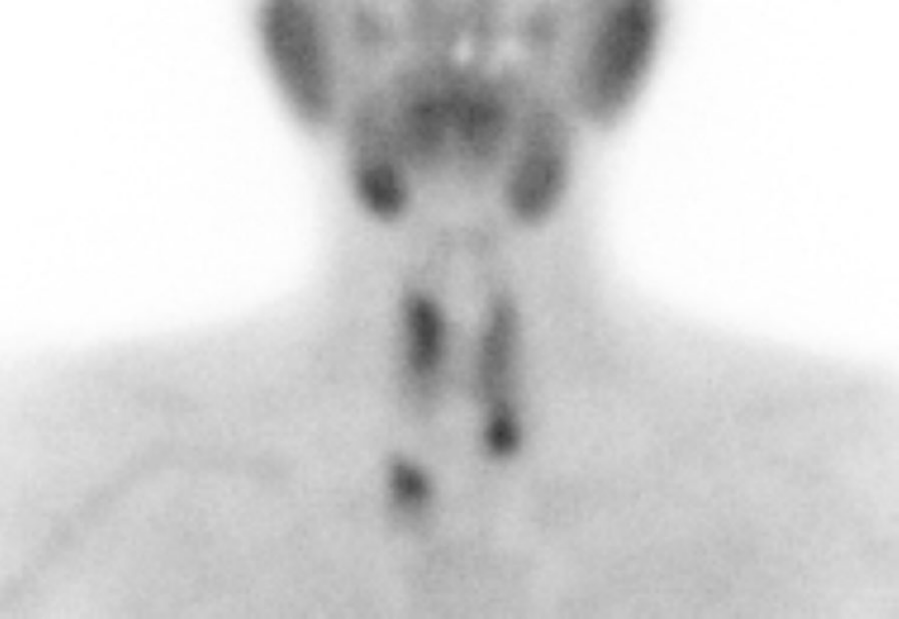
^99m^Tc-methoxyisobutylisonitrile scintigraphy showing increased uptake in the neck region

**Fig. 2:**
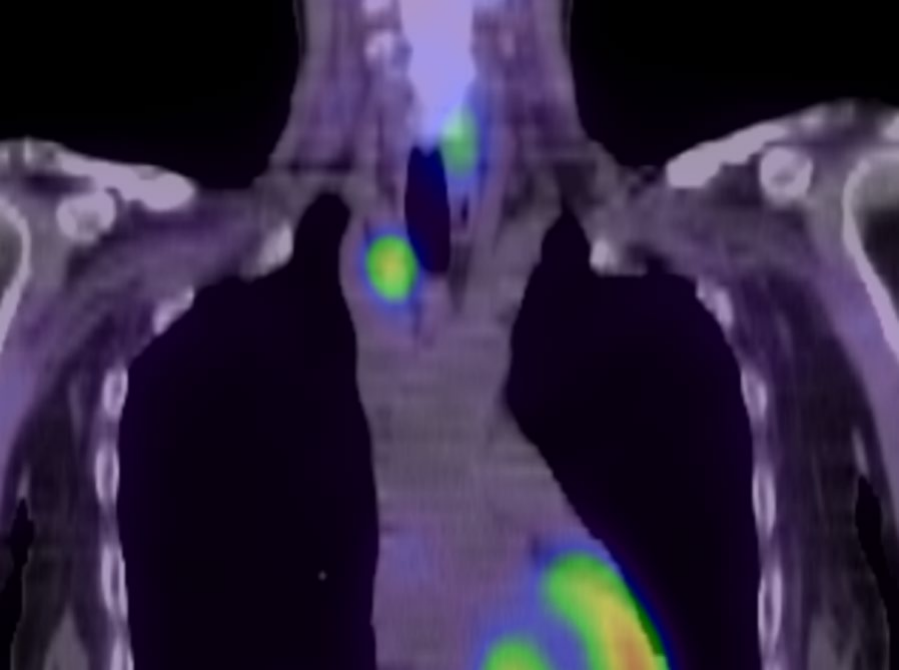
^99m^Tc-methoxyisobutylisonitrile scintigraphy showing increased uptake in the right upper mediastinum after right hemithyroidectomy

**Fig. 3:**
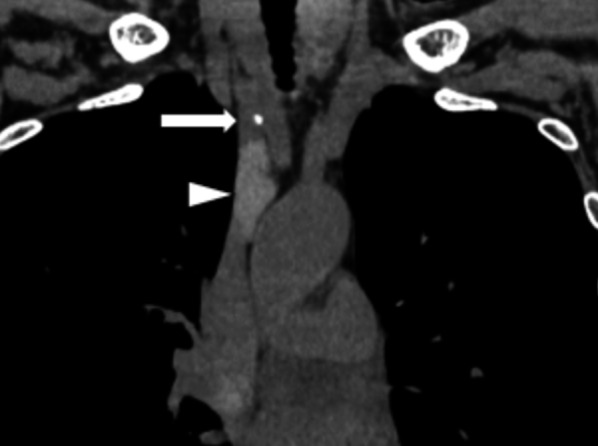
Chest computed tomography showing the right upper mediastinal mass containing a solid part of cephalic lesion (arrow) and a cystic part of caudal lesion (arrowhead). The tumor was located in the anterior side of the trachea and in the posterior side of the superior vena cava

A 53-year-old woman clinically diagnosed with MEN1 was referred to our hospital. She previously underwent laparoscopic distal pancreatectomy for a pancreatic neuroendocrine tumor and presented with a nonfunctional pituitary adenoma that could be followed up, a mediastinal tumor, and asymptomatic hyperparathyroidism. ^99m^Tc-methoxyisobutylisonitrile (MIBI) scintigraphy showing increased uptake in the neck region (Fig. 1). The diagnosis of mediastinal tumor was uncertain; however, after the diagnosis of primary hyperparathyroidism, the patient underwent total parathyroidectomy with autotransplantation of the parathyroid gland on the left forearm and hemithyroidectomy for a thyroid nodule. Four adenomatous parathyroid glands were confirmed in frozen sections. The thyroid tumor was diagnosed as a papillary thyroid carcinoma. Postoperatively, the intact PTH level transiently decreased but returned to the preoperative level within 2 weeks. MIBI scintigraphy was performed again to identify the ectopic parathyroid adenomas (EPAs), which revealed that MIBI had accumulated in the right upper mediastinum (Fig. 2). Chest computed tomography revealed a 45-mm-sized mass behind the superior vena cava (SVC) in the right upper mediastinum as a well-defined solid and cystic lesion (Fig. 3). Magnetic resonance imaging was performed to confirm the absence of tumor invasion into the surrounding tissues and other tumors. Blood tests revealed high levels of intact PTH (103 pg/mL) and Ca (11.4 mg/dL).

**Fig. 4:**
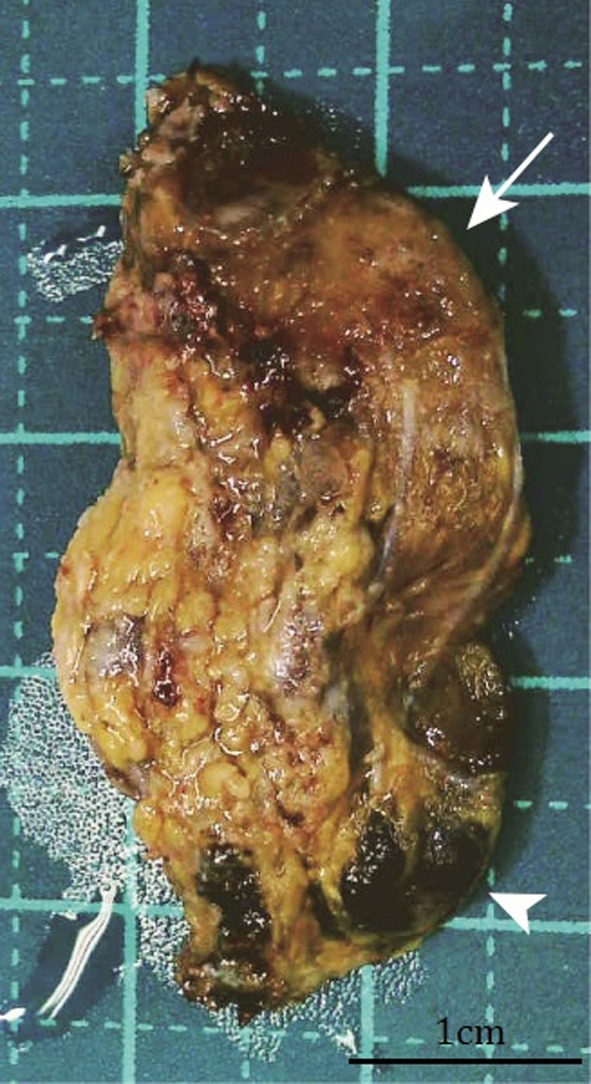
Pathological examination of the mediastinal ectopic parathyroid adenoma. A macroscopic image showing that the tumor is composed of a solid component (arrow) and a cystic component (arrowhead)

**Fig. 5:**
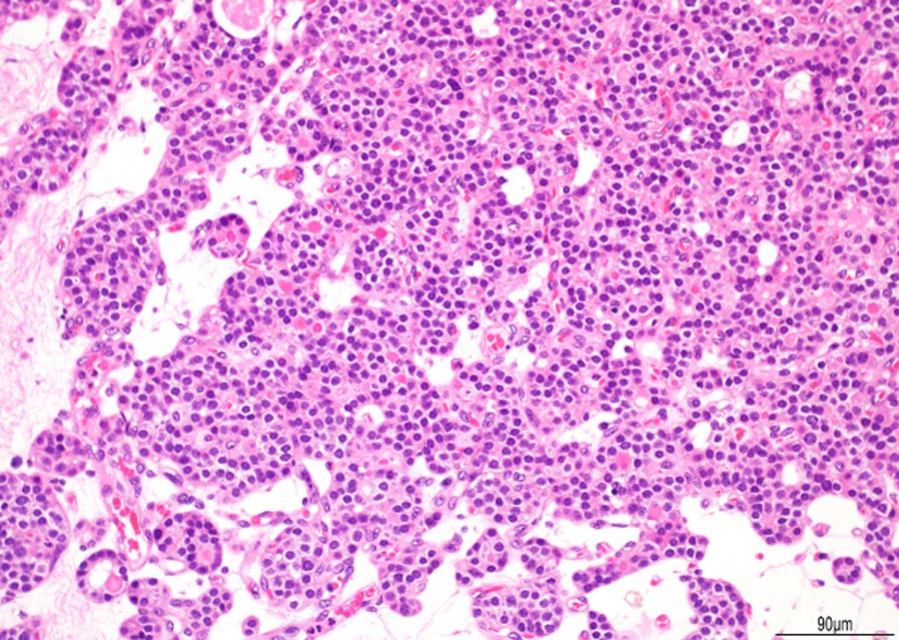
Microscopic image showing that cells with oval nuclei and clear cytoplasm are growing in alveolar or sheet-like forms

Based on various preoperative laboratory and radiological findings, the mediastinal tumor was clinically diagnosed as ectopic parathyroid hyperplasia. RATS was performed using the da Vinci Xi system (Intuitive Surgical Inc., Sunnyvale, CA, USA) with four arms. A robotic endoscope was placed in the seventh intercostal space (ICS) on the middle axillary line, and three robotic operating trocars were placed in the fifth ICS for the right hand, eighth ICS for the left hand, and eighth ICS for the fourth arm. All robotic trocars were 8 mm ports. Fenestrated bipolar forceps, a monopolar spatula, and SynchroSeal (Intuitive Surgical Inc., Sunnyvale, CA, USA) were used. A 12-mm assistant port was also placed in the eighth ICS on the anterior axillary line with carbon dioxide insufflation. The mediastinal tumor was located between the SVC and trachea. The ectopic adenoma was easily detected, and the surgical margin could be clearly identified. The operation time was 76 min, console time was 46 min, and amount of blood loss was minimal. The tumor morphology was consistent with preoperative radiological findings; therefore, it was completely resected using RATS without injuring the capsule. Blood tests performed 1 day postsurgery showed a decrease in calcium level (8.3 mg/dL), and the patient was discharged on the 4th day without postoperative complications. Two weeks after the surgery, the serum intact PTH level decreased to 25.6 pg/mL. The final pathological diagnosis was ectopic parathyroid adenoma with solid and cystic components (Figs. 4, 5).

## Discussion

EPAs are caused by migratory behaviors during embryogenesis. The inferior parathyroid glands, which are derived from the third branchial arch, share their origins with the thymus. Therefore, EPAs can be found in the thymus and anterior or upper mediastinum, even down to the aortopulmonary window. Approximately 20% of the parathyroid adenomas are located in ectopic sites, whereas ectopic mediastinal parathyroid adenomas, which require a thoracic approach, are reported in 1–2% of all cases [2, 3]. In this case, the accumulation on the first MIBI scintigraphy appeared to be in the neck region, and we thought that we could resect all the parathyroid glands using a cervical approach. However, the serum intact PTH level after parathyroidectomy did not decrease to normal; therefore, we performed MIBI scintigraphy again and recognized accumulation in the upper mediastinal region, which revealed that the mediastinal tumor was an ectopic parathyroid gland. In our opinion, the secretion of intact PTH from the parathyroid gland was initially dominant, and intact PTH levels decreased after parathyroidectomy. Intact PTH secretion from the ectopic gland quickly increased, and intact PTH levels were restored.

Complete surgical resection is crucial for successful treatment of hyperparathyroidism. Prior to surgery for EPA, imaging modalities, such as computed tomography, magnetic resonance imaging, and MIBI scintigraphy, indicate the size, morphology, and location of the tumors. One study reported the usefulness of monitoring intraoperative PTH levels to reconfirm complete resection of EPAs [4]. In our patient, after confirming the presence of only one tumor in the upper mediastinum on preoperative radiological examination, the intact PTH levels were not monitored. Using a robot-assisted procedure, we identified and removed well-defined solid and cystic lesions without damaging the tumor capsule. However, if a surgeon is uncertain about the targeted tumor, measurement of intraoperative PTH levels for a more precise surgery would be helpful.

RATS is performed worldwide for thoracic surgery and has several advantages. Robotic instruments have novel characteristics, such as a three-dimensional view, tremor filtering, articulated instruments with 360° rotations, and seven degrees of freedom. Because of these innovations, RATS can be used for meticulous and time-consuming surgical procedures. Several case reports have described robotic-assisted mediastinal parathyroidectomy for hyperparathyroidism [4, 5]. In Japan, RATS has been widely performed for mediastinal and malignant lung tumors, since the cost of the procedure was under public health insurance coverage starting in 2018 [1]. Many thoracic surgeons consider RATS to be more suitable for mediastinal tumors, and RATS has been reported to improve the outcomes of minimally invasive resection of mediastinal tumors [6]. Several authors have reported that seeding of tissues occur during parathyroidectomy in the neck (referred to as “parathyromatosis”) [7–9]. As this is likely the result of traumatic handling of the parathyroid tissue, every effort should be made to remove ectopic parathyroids without compromising tumor integrity [8, 9]. Although preoperative localization of the causative lesion is crucial for curative resection, visibility and maneuverability are also crucial for secure resection. In conclusion, RATS allowed us to achieve a complete and secure resection of the mediastinal EPA.

## Conclusion

Herein, we report a case of a mediastinal EPA that was successfully resected using RATS.

## Data Availability

All data generated or analyzed during this study are included in this published article.

## Abbreviations

PTH: Parathyroid hormone
Ca: Calcium
MEN1: Multiple endocrine neoplasia type 1
RATS: Robot-assisted thoracoscopic surgery
MIBI: ^99M^Tc-methoxyisobutylisonitrile
EPA: Ectopic parathyroid adenoma
SVC: Superior vena cava
ICS: Intercostal space
